# *NLRP3* expression in mesencephalic neurons and characterization of a rare *NLRP3* polymorphism associated with decreased risk of Parkinson’s disease

**DOI:** 10.1038/s41531-018-0061-5

**Published:** 2018-08-15

**Authors:** Katharine M. von Herrmann, Lucas A. Salas, Eileen M. Martinez, Alison L. Young, Joseph M. Howard, Mary S. Feldman, Brock C. Christensen, Owen M. Wilkins, Stephen L. Lee, William F. Hickey, Matthew C. Havrda

**Affiliations:** 1 Department of Molecular and Systems Biology, Geisel School of Medicine at Dartmouth and Dartmouth-Hitchcock Medical Center, Lebanon, NH USA; 2 Department of Epidemiology, Geisel School of Medicine at Dartmouth and Dartmouth-Hitchcock Medical Center, Lebanon, NH USA; 3 Department of Community and Family Medicine, Geisel School of Medicine at Dartmouth and Dartmouth-Hitchcock Medical Center, Lebanon, NH USA; 4 Department of Neurology, The Clinical Neuroscience Institute at Premier Health, Wright State University, Dayton, OH USA; 5 Department of Neurology, Geisel School of Medicine at Dartmouth and Dartmouth-Hitchcock Medical Center, Lebanon, NH USA; 6 Department of Pathology, Geisel School of Medicine at Dartmouth and Dartmouth-Hitchcock Medical Center, Lebanon, NH USA

## Abstract

Neuroinflammation is a well-characterized pathophysiology occurring in association with the progression of Parkinson’s disease. Characterizing the cellular and molecular basis of neuroinflammation is critical to understanding its impact on the incidence and progression of PD and other neurologic disorders. Inflammasomes are intracellular pro-inflammatory pattern-recognition receptors capable of initiating and propagating inflammation. These cellular complexes are well characterized in the innate immune system and activity of the NLRP3 inflammasome has been reported in microglia. NLRP3 inflammasome activity has been associated with Alzheimer’s disease, and recent reports, from our laboratory and others, indicate that *Nlrp3* is required for neuroinflammation and nigral cell loss in animal models of PD. *NLRP3* has not yet been characterized in PD patients. Here we characterize *NLRP3* in PD using immunohistologic and genetic approaches. Histologic studies revealed elevated *NLRP3* expression in mesencephalic neurons of PD patients. Analysis of exome sequencing data for genetic variation of *NLRP3* identified multiple single-nucleotide polymorphisms (SNPs) including rs7525979 that was associated with a significantly reduced risk of developing PD. Mechanistic studies conducted in HEK293 cells indicated that the synonymous SNP, *NLRP3* rs7525979, alters the efficiency of *NLRP3* translation impacting NLRP3 protein stability, ubiquitination state, and solubility. These data provide evidence that dopaminergic neurons are a cell-of-origin for inflammasome activity in PD and are consistent with recent animal studies, suggesting that inflammasome activity may impact the progression of PD.

## Neuroinflammation: a desirable genetic variant

A genetic variant of a protein that mediates neuroinflammatory processes reduces the risk of developing Parkinson’s disease (PD). Previous studies have shown that NLRP3 contributes to the progression of Alzheimer’s disease and PD in animal models. Matthew Havrda at the Geisel School of Medicine at Dartmouth, New Hampshire, USA, and colleagues found high levels of NLRP3 in degenerating dopaminergic neurons in the brains of patients with PD. Interestingly, analyses of genetic sequences coding NLRP3 obtained from the Parkinson’s Progression Markers Initiative revealed an NLRP3 variant associated with *a significantly decreased risk of PD*. This effect might be due to its reduced activity as the NLRP3 variant formed insoluble aggregates in cells. Further understanding the role of NLRP3 in neurodegenerative diseases could aid the development of novel neuroinflammatory inhibitors.

## Introduction

Inflammation is a characteristic of both normal aging and progressive neurodegenerative disorders of the elderly.^1,2^ Pathologic examination of postmortem tissues,^3^ animal modeling,^4^ genome-wide association studies,^5^ and epidemiologic studies^6^ have related neuroinflammation with Parkinson’s disease (PD).^7–9^ Inflammasomes are multi-protein complexes that mediate inflammation in response to both pathogens and sterile inflammatory triggers. The function of inflammasomes is best characterized in myeloid lineages where inflammasome activation results in caspase 1-dependent activation and secretion of interleukin (IL)-1β.^10^ Inflammasome complexes are defined by the specific cytosolic nucleotide binding and oligomerization domain (NOD)-like receptor (NLR) family member that they contain. Proteins of the pattern-recognition NLR family share homology in a highly conserved NACHT domain along with an n-terminal pyrin domain and a c-terminal leucine-rich repeat domain.^11^ Expression of inflammasome proteins is observed in the central nervous system (CNS), typically in monocytes and microglia responding to acute infection or cell loss as occurs during neurodegeneration.^12^ Although poorly understood, expression of the *NLRP1*,^13^ *AIM2*,^14^ and *NLRP3*^15^ NLRs has been detected in neurons. These findings suggest that inflammasomes may function in neuronal cells of the CNS providing a novel mechanism by which neurons may initiate or influence inflammation.

The NLRP3 inflammasome is implicated in the pathogenesis of Alzheimer’s disease (AD),^16^ but a role for *NLRP3* in PD has not been characterized. Our recent animal studies indicate that mice lacking *Nlrp3* are resistant to the development of PD symptomatology resulting from exposure to the pesticide rotenone.^17^ Our findings are consistent with the reports of others who have observed that mice lacking either *Nlrp3* or the key inflammasome effector *Caspase 1* (*Casp1*) are resistant to nigral cell loss resulting from acute exposure to the neurotoxin MPTP.^18,19^ Further, Mao et al. recently published findings supporting a pathogenic role for the NLRP3/capase-1/IL-1β axis in the 6-OHDA PD rat model.^20^ The potential role for *NLRP3* in PD is also supported by in vitro studies that describe Nlrp3 inflammasome activation by mitochondrial reactive oxygen species (ROS) in monocytes^21^ and by pathologic α-synuclein in cultured glia,^22^ two physiologically relevant inflammatory triggers associated with the progression of both the idiopathic and monogenic forms of PD.^23–26^ Although not yet characterized in PD, it is noteworthy that dopaminergic (DA) neurons may provide a microenvironment in which inflammasomes could be activated to initiate or propagate neuroinflammation. DA neurons are subject to inflammasome-related cellular stressors, including synuclein proteinopathy,^27^ oxidative stress associated with dopamine metabolism,^28^ and mitochondrial stress associated with genetic alterations or exposure to environmental toxins.^21,29,30^ Understanding whether the NLRP3 inflammasome functions in DA neurons in response to PD-associated sterile inflammatory triggers would be of broad interest, as determining how DA neurons could initiate chronic neuroinflammation in PD may shed light on the cellular and molecular mechanisms underlying the earliest stages of disease progression.

Targeting neuroinflammation to treat progressive neurologic disorders is a rational neuroprotective strategy but to realize the potential of such approaches, the cellular and molecular pathways critical for disease-associated neuroinflammation must be identified.^11^ Our recent report indicates that *Nlrp3* is required for the development of inflammatory symptoms and neurodegeneration in a mouse model of PD.^17^ Here we evaluated the expression of *NLRP3* in human tissues obtained from histopathologically confirmed cases of idiopathic PD and control subjects. We found elevated *NLRP3* expression in surviving pigmented neurons of the mesencephalon in tissues obtained from PD patients, suggesting that inflammasomes in DA neurons may contribute to PD-associated neuroinflammation. This potential etiologic contribution of *NLRP3* to PD led us to evaluate the genetic variation in *NLRP3* within exome sequencing data obtained from the Parkinson’s Progression Markers Initiative (PPMI). We identified multiple single-nucleotide polymorphisms (SNPs) clustered in the highly conserved *NLRP3* NACHT domain. One variant, synonymous SNP rs7525979, was associated with a significantly reduced risk of developing PD. This association also differentiated PD patients from a population of SWEDD (scans without evidence of dopaminergic deficit) patients. Evaluating the impact of polymorphism *NLRP3* rs7525979 in HEK293 cells, we provide characterization of this synonymous variant impacting the NLRP3 protein life cycle.

## Results

### NLRP3 expression in human mesencephalic tissues and neuronal cultures

**Fig. 1:**
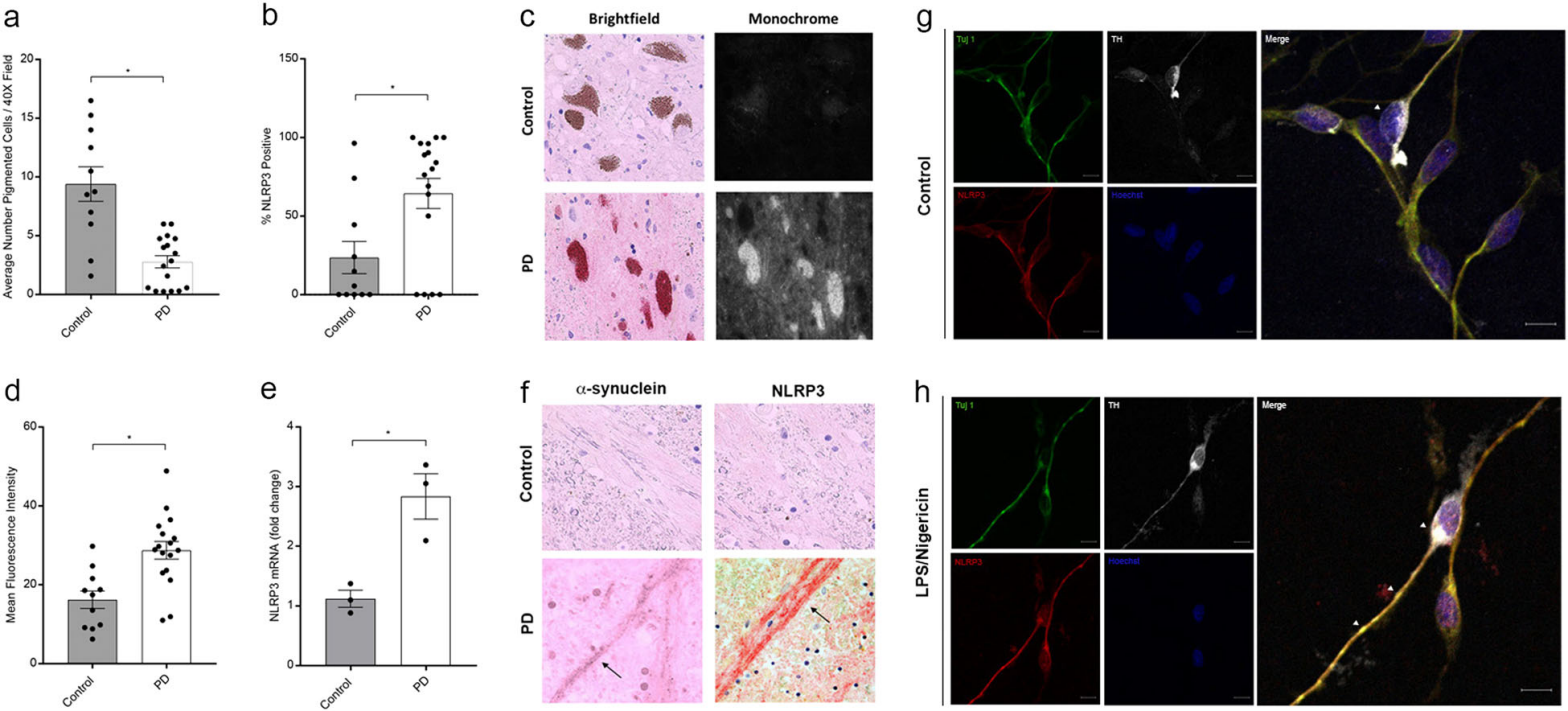
Elevated *NLRP3* expression in human PD tissues. **a** Estimations of DA neuron density were determined by counting pigmented cell bodies across six 40× fields within the human substantia nigra in histologic sections obtained from control (*n* = 11) and PD patients (*n* = 17). The average number of pigmented neurons was significantly reduced in sections obtained from PD patients as compared with controls (*P < 0.001, two-tailed *t* test). **b** The percentage of NLRP3-positive cells determined in NLRP3 stained histologic sections. The average percentage of neuromelanin pigmented NLRP3-positive neurons was increased in sections obtained from PD patients (50 neurons counted per section, *P = 0.0413, two-tailed *t* test). **c** Immunohistologic sections obtained from the mesencephalon of controls (upper panels) and PD patients (lower panels) stained using anti-NLRP3 antibodies were viewed using both brightfield (left panels) and fluorescent excitation (595/Texas red filter and photographed in monochrome right panels). We used this method to assure specificity, as fluorescence specifically excites the fast red chromagen (lower right panel) and not endogenous neuromelanin (upper right panel). **d** Computer-assisted quantitation of mean per cell fluorescence was determined using a line profile approach (ImagePro). Mean NLRP3 immunofluorescence was elevated in cells of PD patients as compared with controls (12 representative neurons measured per section, control subjects (*n* = 11), PD patients (*n* = 17), *P < 0.001, two-tailed *t* test). **e** Total mRNA was isolated from representative cryopreserved mesencephalic tissues obtained from control (*n* = 3) and PD patients (*n* = 3), reverse transcribed, and analyzed using real-time PCR. *NLRP3* mRNA was elevated in PD samples as compared with controls (*P < 0.01, two-tailed *t* test). Error bars represent s.e.m. **f** Immunohistologic sections obtained from the human mesencephalon of control subjects and PD patients were stained with anti-α-synuclein antibodies and anti-NLRP3 antibodies. No immunoreactivity was observed in DA fibers of the ventral substantia nigra in control samples. Lewy neurites were observed in sections obtained from PD patients immunostained using anti-α-synuclein antibodies (left panel, indicated with black arrow). NLRP3 immunoreactive neurites were only observed in PD patient samples (right panel, indicated with black arrow). **g** Confocal images of differentiated LUHMES cells treated with vehicle, fixed, immunostained with anti-Tuj1, anti-tyrosine hydroxylase (TH), and anti-NLRP3 antibodies, and counterstained with Hoechst dye. Scale bar represents 20 μM. **h** Differentiated LUHMES cells treated with LPS followed by Nigericin fixed and stained as described in **g**. Arrows indicate TH-positive neurons expressing NLRP3. Fluorescence intensity of Tuj1 and NLRP3 was measured in the cell body and axons from untreated and treated cells. NLRP3 fluorescence, normalized to Tuj1, was significantly greater in both the axons and cell bodies of LUHMES cells treated with LPS and Nigericin. Axons: mean intensity treated of 0.8643 ± 0.02401 vs. untreated 0.6256 ± 0.05577 (*P* = 0.0010, *t* test, *n* = 10 per image). Cell bodies: mean intensity treated of 0.8297 ± 0.02522 vs. untreated 0.6542 ± 0.02936 (*P* = 0.0003, *t* test, *n* = 10 per image)

To characterize the role of *NLRP3* in PD, we examined postmortem tissues obtained from the mesencephalon of PD patients (*n* = 17) and control subjects (*n* = 11). We compared the percentage of NLRP3 positive-cell bodies observable in histologic sections of mesencephalic tissues. As expected, we observed significantly fewer DA neurons in mesencephalic tissues from PD patients compared with healthy controls (Fig. 1a). While there were fewer DA neurons in the mesencephalon in PD patient tissues, a significantly greater percentage of surviving neurons were NLRP3-positive in tissues obtained from PD patients relative to controls (Fig. 1b). We used computer-assisted image analysis to quantify the intensity of NLRP3 immunoreactivity in DA cell bodies in mesencephalic tissues and observed a significant increase in NLRP3 staining intensity in surviving DA neurons in PD as compared with healthy donors (Fig. 1c, d). We also observed elevated levels of *NLRP3* mRNA in PD patient mesencephalic brain homogenates obtained from PD patients as compared with the levels observed in control samples (Fig. 1e). In addition to pigmented cell bodies, we analyzed NLRP3 immunoreactivity in Lewy neurites, non-pigmented, α-synuclein immunoreactive neuronal processes observed in PD.^31^ Staining for α-synuclein, we confirmed the presence of these processes only in histologic sections obtained from PD patients (Fig. 1f, left panel). In adjacent sections, we detected robust NLRP3 immunoreactivity within Lewy neurites only in sections obtained from PD patients (Fig. 1f, right panel). Seeking evidence of inflammasome activation associated with elevation in NLRP3 protein, we evaluated tissues obtained from PD patients and controls for the NLRP3 inflammasome target CASP1. We identified increased CASP1 immunoreactivity in histologic sections of the mesencephalon obtained from PD patients as compared with tissues from controls (Supplementary Figure 1A). Immunoreactivity appeared throughout the neuropil within the mesencephalon. High magnification revealed punctate CASP1 immunoreactivity only in tissues obtained from PD patients (Supplementary Figure 1A, right panels). We extended these histologic findings of elevated CASP1 in extracts obtained from freshly cryopreserved tissues using sodium dodecyl sulfate-polyacrylamide gel electrophoresis (SDS-PAGE). These studies verified an increase in the level of the 45 kDa CASP1 zymogen in PD-derived tissues as compared with controls (Supplementary Figure 1B). CASP1 activation is characterized by cleavage of the zymogen into p20 and p10 subunits that subsequently interact to form the active enzyme. We evaluated extracts obtained from available cryopreserved tissues for these low-molecular-weight proteins that are indicative of CASP1 activation. We detected the p10 subunit in three of the six PD extracts that we analyzed and did not detect such bands in the extracts obtained from four controls (Supplementary Figure 1C). These data indicate that CASP1 levels are elevated within the degenerating mesencephalon in PD and that activated CASP1 is detectable in a subset of PD patients.

In addition to our histologic findings in human PD tissues, we conducted dual-labeling immunocytochemistry experiments using the differentiated LUHMES cell model of human DA neurons.^32^ We observed induction of NLRP3 in neurons following treatment with inflammasome-activating agent Nigericin following lipopolysaccharide (LPS) priming in Tuj1-positive neuronal cells. Among these neurons were a subset of cells with advanced morphology clearly co-expressing NLRP3 along with tyrosine hydroxylase (Fig. 1g, h). We also observed NLRP3 expression in both undifferentiated and differentiated SH-SY5Y cells (Supplementary Figure 2A) and measured a statistically significant increase in CASP1 activation upon treatment with LPS and Nigericin (Supplementary Figure 2B) in differentiated cultures. Together, our histologic and biochemical findings indicate elevated *NLRP3* transcript and protein expression in the degenerating mesencephalon of PD patients as compared with tissues obtained from control donors and identify DA neurons as a potential cellular origin for inflammasome activity in PD.

### *NLRP3* genetic variant associated with a reduced risk of PD

**Fig. 2:**
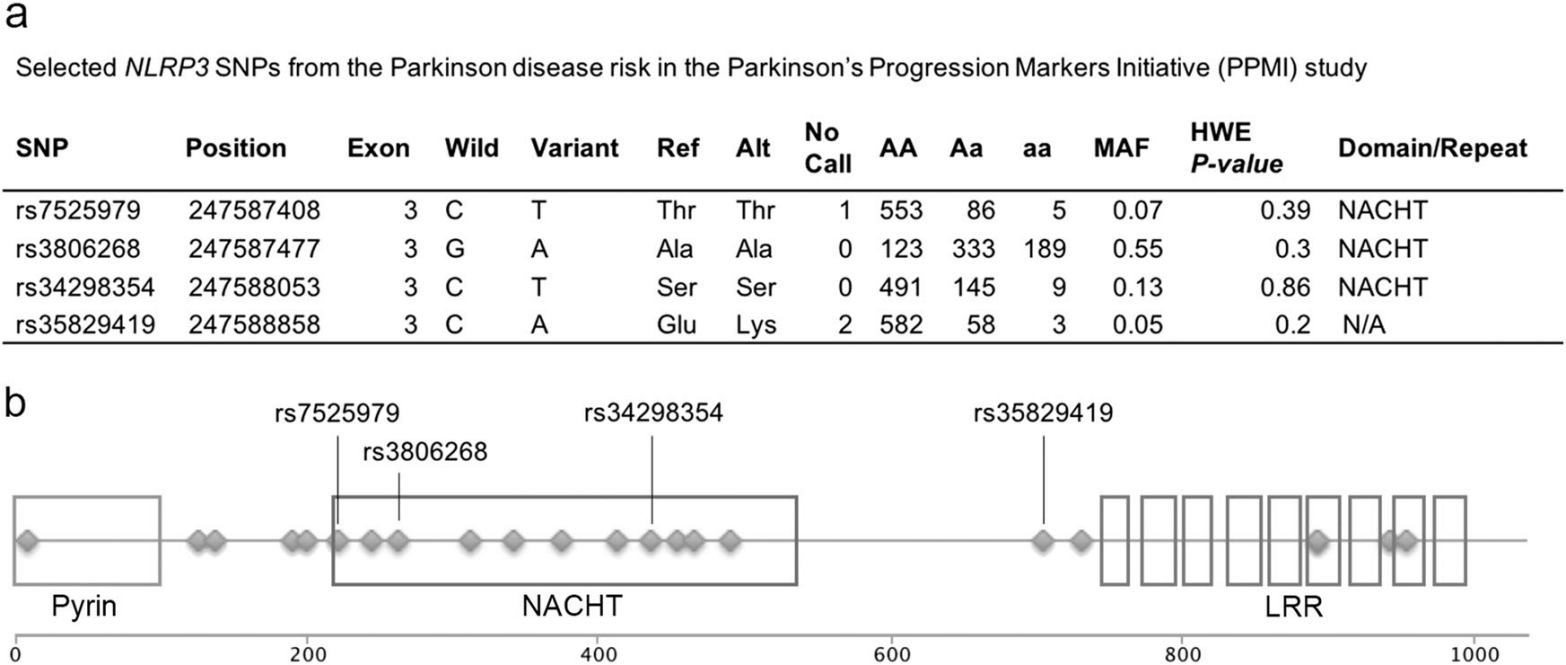
Genetic variation of *NLRP3*. **a** Whole-exome sequences of individuals in the PPMI database (*n* = 644: 402 PD patients, 60 SWEDD, and 182 controls) were assessed. A total of 22 SNPs in *NLRP3* were identified. Four SNPs with an MAF ≥ 5% in this cohort were selected for further analysis rs7525979, rs3806268, rs34298354, and rs35829419. Table header abbreviations: SNP single nucleotide polymorphism, Position nucleotide position within the genome, Broad Institute, Exon location of SNP, Wild WT nucleotide, Variant variant nucleotide, Ref amino acid coded for by WT nucleotide, Alt amino acid coded for by inclusion of variant nucleotide, MAF minor allele frequency, HWE *P* value Hardy-Weinberg Equilibrium analysis *P* value, Domain/Repeat location of SNP within protein. **b** Schematic representation of the *NLRP3* gene structure. *NLRP3*, located on the long arm of chromosome 1, codes for a multi-functional NOD-like receptor (NLR) protein containing a highly conserved NACHT domain along with an n-terminal pyrin domain and a c-terminal leucine-rich repeat domain. The protein is comprised of 1036 amino acids and has a molecular mass of 118 kDa. Three of the four NLRP3 SNPs identified in this study that have a MAF ≥ 5% are located in the NACHT domain. All SNPS with a MAF ≥ 5% are indicated with respective Reference SNP numbers. All 22 NLRP3 SNPs identified in the PPMI database are indicated with a diamond along the protein diagram

As *NLRP3* has not been previously characterized in PD patients, we sought additional evidence of a role for *NLRP3* by assessing its genetic variation in PD patients and controls. We used exome sequence data obtained through the PPMI to identify coding region SNPs (*n* = 644: 402 PD patients, 60 SWEDD, and 182 controls).^33^ Twenty-two SNPs in *NLRP3* were available in the sequencing data, four SNPs had a minor allele frequency (MAF) ≥ 5% and were selected for further analysis (rs7525979, rs3806268, rs34298354, and rs35829419) (Fig. 2a). Three of the four SNPs selected for further analysis are located in the catalytic NACHT domain of the NLRP3 protein (Fig. 2b). We modeled the relationship of the four SNPs with PD risk with dominant unconditional logistic regression adjusted for subject age and sex. The C-to-T variant of rs7525979 was associated with a significantly decreased risk of PD, odds ratio (OR) [95% confidence interval (CI)]: 0.55 [0.34–0.89] (Table 1). Results from an additive inheritance model were consistent with this finding (Supplementary Table 1). As a sensitivity analysis, we restricted our models to subjects self-reported as “white” race *n* = 613 (95%), and the dominant model showed similar results OR [95% CI]: 0.58 [0.34–0.99]. Neither the C-to-T variant of rs7525979 nor the other *NLRP3* SNP variants (data not shown) were significantly related to SWEDD (Table 2). The C-to-T variant rs7525979, associated with a significantly decreased risk of PD (Table 1), is a synonymous SNP and therefore is not expected to alter the amino acid sequence of NLRP3. These data indicate that rs7525979 warrants further investigation to determine the mechanism by which its presence could alter the risk of PD.

**Table 1:** Parkinson disease risk in the Parkinson’s Progression Markers Initiative (PPMI) study associated with the *NLRP3* SNP rs7525979

	Cases *n* = 402, *n* (%)	Controls *n* = 182, *n* (%)	OR (95% CI)	*P* value
Age, mean (sd)	62.2 (9.7)	61.6 (11.0)	1.006 (0.99–1.02)	0.53
Sex				
Male	260 (64.7)	116 (63.7)	1.0 (Reference)	
Female	142 (35.3)	66 (36.3)	0.94 (0.65–1.36)	0.73
rs7525979 (C/C)				
C/C	357 (88.8)	148 (81.3)	1.0 (Reference)	
C/T+T/T	45 (11.2)	34 (18.7)	0.55 (0.34–0.89)	<0.02

Model adjusted for all variables in the table

**Table 2:** Scans without evidence of dopaminergic deficit (SWEDD) risk in the Parkinson’s Progression Markers Initiative (PPMI) study associated with the *NLRP3* SNP rs7525979

	SWEDD subjects *n* = 60, *n* (%)	Controls *n* = 182, *n* (%)	OR (95% CI)	*P* value
Age, mean (sd)	61.4 (10.1)	61.6 (11.0)	0.999 (0.98–1.03)	0.95
Sex				
Male	37 (61.6)	116 (63.7)	1.0 (Reference)	
Female	23 (38.3)	66 (36.2)	1.10 (0.59–2.00)	0.77
rs7525979 (C/C)				
C/C	48 (80.0)	148 (81.3)	1.0 (Reference)	
C/T+T/T	12 (20.0)	34 (18.7)	1.10 (0.51–2.24)	0.81

Model adjusted for all variables in the table

### Synonymous *NLRP3* variant rs7525979 impacts translation efficiency in vitro

**Fig. 3:**
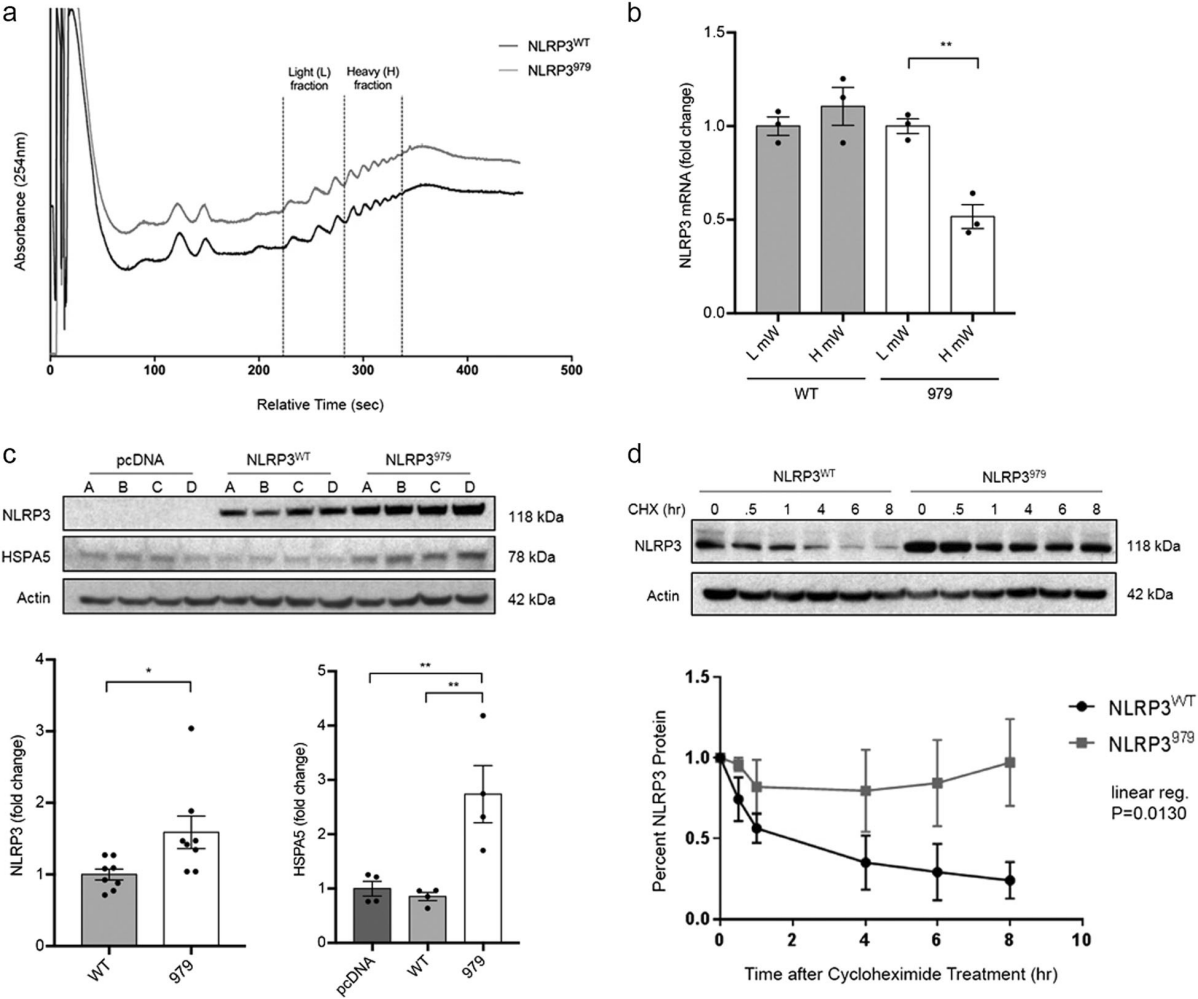
*NLRP3* SNP rs7525979 impacts NLRP3 protein life cycle in vitro. Four independent polyclonal HEK293 cell lines were generated to express *NLRP3*^*WT*^, *NLRP3*^*979*^, or empty vector control. **a** Polysome profiling analysis comparing cells expressing *NLRP3*^*WT*^ or *NLRP3*^*979*^ (absorbance was measured at 254 nm, tracings are offset to improve visualization). **b** Relative *NLRP3* mRNA transcript levels, quantified by real-time PCR, in the light and heavy polysome fractions as indicated in **a**. Representative data shown, assayed in triplicate, two biologic repeats. (*P* = 0.0011, one-way ANOVA). **c** Representative immunoblots indicating steady-state levels of NLRP3 and HSPA5 in four independently selected polyclonal cultures as measured following gel fractionation using SDS-PAGE. NLRP3^979^ expression was significantly greater than NLRP3^WT^ expression across multiple independent cell lines (*P < 0.05, *t* test). HSPA5 expression was significantly greater in cultures expressing NLRP3^979^ compared to cells expressing NLRP3^WT^ (**P < 0.01, one-way ANOVA). **d** NLRP3 protein stability experiments were conducted with cycloheximide (CXH) treatment over 8 h, followed by SDS-PAGE and immunoblotting. Representative immunoblotting of NLRP3 in CHX treated cells over 8 h. Linear regression analysis was performed using the GraphPad Prism software. (*P* = 0.0130, *n* = 3 biologic replicates). Protein loading in all immunoblotting experiments was controlled by normalizing to beta actin. Error bars in **b**, **c** represent s.e.m. Blots are representative, derived from the same experiment, processed in parallel, and replicated as described

To investigate the impact of the rs7525979 variant on the function of the NLRP3 inflammasome, we generated stable HEK293 cell lines expressing either wild-type *NLRP3* (*NLRP3*^*WT*^) or *NLRP3* harboring the rs7525979 C-to-T variant (*NLRP3*^*979*^). We evaluated transcript levels across independently derived HEK293 cell lines and found no evidence of alterations in mRNA levels resulting from the presence of the rs7525979 SNP (data not shown). Although synonymous SNPs do not impact the amino acid sequence, their presence has the potential to impact translation efficiency.^34^ Since the rs7525979 harboring ACT codon is relatively rare compared with the wild-type ACC codon (12.8/1000 compared with 19.2/1000, respectively), we evaluated the efficiency of translation using polysome-profiling techniques. We stabilized ribosome–mRNA polysomes using cyclohexamide and evaluated ribosome load using gradient fractionization (Fig. 3a) followed by real-time PCR to infer translational efficiency.^35^ We observed that the mRNA harboring the rs7525979 variant consistently migrated with lower molecular weight polysomes as compared with the *NLRP3*^*WT*^ transcript (Fig. 3b), indicating that the translation efficiency of *NLRP3*^*979*^ mRNA was altered as compared with that of *NLRP3*^*WT*^ mRNA.

### Variant rs7525979 alters NLRP3 protein stability, ubiquitination, and solubility in vitro

We next characterized the NLRP3 protein life cycle in HEK293 cells expressing either *NLRP3*^*WT*^ or *NLRP3*^*979*^. SDS-PAGE and immunoblotting in four independently selected stable polyclonal cell populations expressing either *NLRP3*^*WT*^ or *NLRP3*^*979*^ revealed elevated levels of NLRP3 protein expression in all four cell lines expressing *NLRP3*^*979*^ as compared with those expressing *NLRP3*^*WT*^ (Fig. 3c). We analyzed candidate genes known to be involved in the regulation of protein translation and folding and found consistent upregulation of the molecular chaperone HSPA5 (Fig. 3c), a heat-shock protein with well-characterized functions in monitoring and maintaining protein folding and turnover.^36^ These findings led us to predict that NLRP3 protein may be accumulating in the cell as a result of decreased translation efficiency resulting from the presence of the synonymous variant rs7525979.

**Fig. 4:**
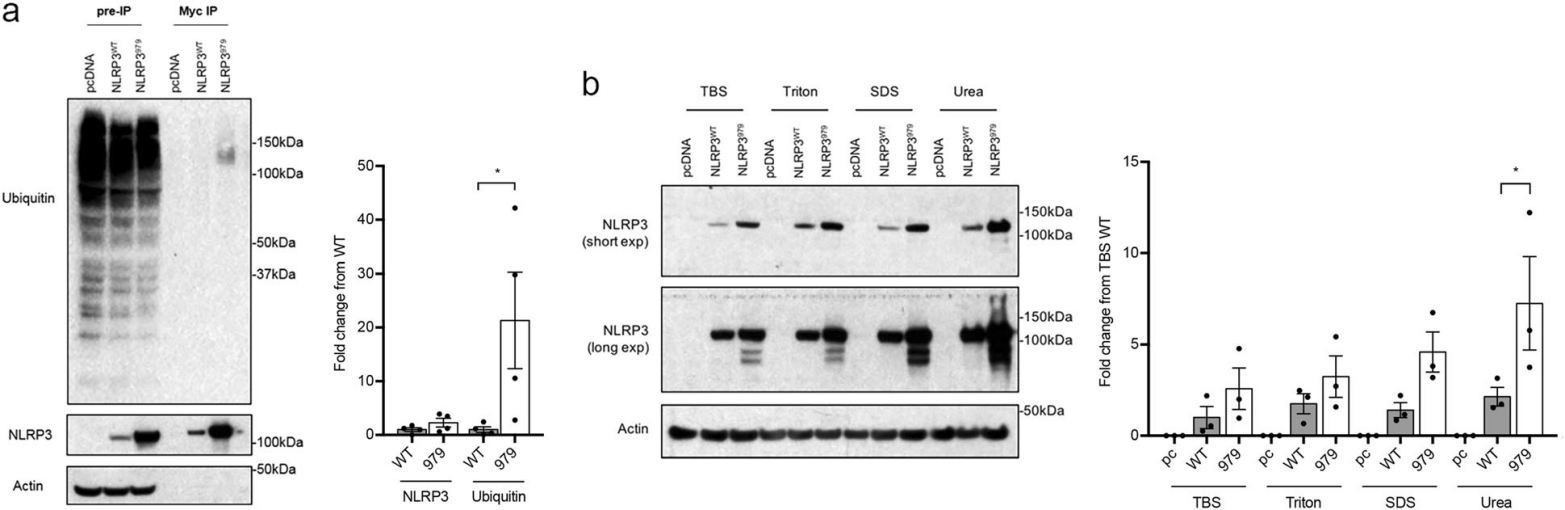
*NLRP3* SNP rs7525979 impacts NLRP3 protein ubiquitination and solubility. **a** Myc-tagged NLRP3 immunoprecipitation assay followed by SDS-PAGE and immunoblotting with anti-ubiquitin antibodies (*n* = 4 biologic replicates). Ubiquitin protein expression normalized to NLRP3, previously normalized to actin. NLRP3^979^ protein was bound to significantly more ubiquitin proteins (*P < 0.05, one-way ANOVA). **b** To assess protein solubility, cell lysates were harvested in TBS buffer, ultracentrifuged to separate insoluble proteins, and then re-suspended in increasingly stringent buffers (Triton, SDS, and Urea, respectively) interspersed by repeated ultracentrifugation steps. Cell lysates were separated by SDS-PAGE and *NLRP3* expression was detected with immunoblotting (*n* = 3 biologic replicates). Significantly more *NLRP3*^*979*^ was detected in the Urea fraction when compared to *NLRP3*^*WT*^ proteins (*P < 0.05, one-way ANOVA). Protein loading in all immunoblotting experiments was controlled by normalizing to beta actin. Error bars represent s.e.m. Blots are representative, derived from the same experiment, processed in parallel, and replicated as described

To test our prediction of NLRP3^979^ accumulation, we evaluated the stability of NLRP3 protein in HEK293 cells expressing either *NLRP3*^*WT*^ or *NLRP3*^*979*^. NLRP3 encoded by *NLRP3*^*979*^ had a significantly increased stability than that encoded by *NLRP3*^*WT*^ (Fig. 3d). We next investigated the ubiquitination state of NLRP3 protein by immunoprecipitating NLRP3 in cell lines expressing either *NLRP3*^*WT*^ or *NLRP3*^*979*^. *NLRP3*^*979*^-encoded NLRP3 was found to be more ubiquitinated than *NLRP3*^*WT*^-encoded protein (Fig. 4a). Misfolded proteins are often sequestered in the form of polyubiquitinated insoluble aggregates.^37^ Therefore, to assess the relative solubility of NLRP3, we conducted a serial ultracentrifugation assay re-suspending insoluble fractions in increasingly stringent buffers. NLRP3 encoded by *NLRP3*^*979*^ was less soluble than that encoded by *NLRP3*^*WT*^, as more *NLRP3*^*979*^-encoded protein was present in the Urea buffer fraction (Fig. 4b). This series of experiments supports a model in which the presence of synonymous variant rs7525979 deregulates *NLRP3* translation-altering aspects of the NLRP3 protein life cycle, including protein stability, ubiquitination state, and solubility.

## Discussion

Postmortem analyses clearly indicate that neuroinflammation is associated with PD^3^ and many of the environmental and genetic risk factors postulated for PD^29,30,38^ are believed to impact the inflammatory state of the brain.^39^ Inflammasomes have only recently been identified in the CNS^40^ and our animal studies suggest that they might be a rational target for anti-inflammatory intervention to treat neurologic disorders associated with inflammation.^11^ Here we conduct histologic and genetic studies in PD patients to determine whether *NLRP3* may play a role in the onset and progression of disease. Histologic studies identify expression of *NLRP3* in DA neurons of the human brain and provide evidence of enhanced neuronal NLRP3 immunoreactivity in tissues obtained from PD patients relative to control subjects. Genetic studies in the PPMI exome sequence dataset identify multiple polymorphisms in *NLRP3*, with one SNP statistically associated with a reduced risk of PD. Further analysis of rs7525979 in vitro demonstrates that this synonymous SNP can elicit changes in the life cycle of NLRP3 protein, ultimately resulting in the accumulation of ubiquitinated, insoluble intracellular protein aggregates. Together, these studies support previously published findings from our laboratory and others,^17–19^ indicating an important role for *NLRP3* in the progression of PD.

We report detection of *NLRP3* expression in DA neurons in PD patients, a finding related to a recent report by Zhang et al., who found expression of the canonical inflammasome targets IL-1β and IL-18 in cerebrospinal fluid obtained from PD patients.^15^ These findings of neuronal inflammasome expression in human tissues extend previous reports that identified neuronal *NLRP1*,^13^ *AIM2*,^14^ and *NLRP3*^15^ expression and function in rodents.^12^ Collectively, these studies suggest that, in neurons, inflammasome proteins may be an important component of the cellular stress response. In the context of PD-associated cellular stressors, both mitochondrial dysfunction and misfolded protein accumulation, two pathological hallmarks of PD, have been reported to activate inflammasome proteins.^22,41^ Specifically, many of the causal mutations in familial PD result in deregulation of mitochondrial function. Misfolded α-synuclein, one of the main components of Lewy bodies in PD, has been observed to activate the NLRP3 inflammasome in cultured microglia. Our identification of enhanced *NLRP3* expression in neurons is of interest because prior studies of the NLRP3 inflammasome in the CNS have primarily focused on microglia. Neuron–glia interactions are of particular importance in the progression of PD^11^ and cytosolic stress sensors like the NLRP3 inflammasome are compelling candidates for mediating such interactions based on the potential for release of diffusible factors during the process of inflammasome activation and pyroptotic cell death.^42^ It is intriguing to consider the possibility that DA neurons may utilize the inflammasome platform to sense intracellular sterile triggers such as ROS and misfolded proteins and then employ inflammasome-dependent cytokine intermediates to activate local glial cells. Such a process is likely to unfold at the earliest stages of PD and may help to explain how CNS stressors including environmental toxicants could increase the risk of developing PD.

*NLRP3* polymorphisms are associated with multiple human inflammatory disorders, including the cryopyrin-associated periodic syndromes (CAPS) spectra,^43^ inflammatory bowel disease,^44^ primary gouty arthritis,^45^ and psoriatic idiopathic juvenile arthritis.^46^ Recent reports identify polymorphisms in *NLRP3* in patients with late-onset AD in a Han Chinese population where the SNP rs35829419 was associated with a reduced risk of developing AD.^47^ In this study, AD-associated SNPs with an MAF ≥ 5% were identified only within non-coding regions. To our knowledge, no studies have identified polymorphisms with an MAF ≥ 5% associated with neurologic disorders in *NLRP3*-coding regions nor has the *NLRP3* gene structure been examined specifically in individuals with PD. Although PD most often occurs sporadically, increasing evidence points to underlying genetic predispositions that can alter the incidence and progression of PD.^24,26,48,49^ PPMI was initiated to expand our understanding of genetic variation occurring in association with PD and is unique in its inclusion of exome sequence data obtained from patients with SWEDD along with data obtained from PD patients and controls.^33^ We took advantage of the PPMI exome sequence data to evaluate genetic variation in *NLRP3*. In these studies, we evaluated *NLRP3* SNPs with an MAF ≥ 5%. Three of the four SNPs we identified with an MAF ≥ 5% were located in the NACHT domain (Fig. 2). The catalytic NACHT domain that was originally identified during analysis of programmed cell death-related proteins is encoded by exon 3 in *NLRP3* and is conserved in animals, fungus, and bacteria.^50^ We also observed a distinct concentration of less frequent polymorphisms in this domain as compared with other regions of *NLRP3* (Fig. 2b). Our findings of multiple polymorphisms within the *NLRP3* NACHT domain are consistent with *NLRP3* mutations associated with CAPS syndromes that also frequently occur in exon 3.^51^ We identified one synonymous SNP in the *NLRP3* NACHT domain, rs7525979, statistically associated with a reduced risk of PD. These findings in the PPMI dataset should be treated with caution because the accuracy of exome sequence data is <100%.^52^ To overcome limitations in our candidate gene discovery results, we also interrogated the PDGene dataset.^7^ Although in the PPMI population the minor allele frequency of the variant was 0.12 (and 0.09 for the “white” self-reported race), this variant was rare in previous meta-analysis^7^ where only 0.03 of the CEU subjects were carriers. Although the direction of effect reported in PDgene is consistent with our results, it did not reach statistical significance, potentially due to reduced imputation accuracy for rare variants. Based on these findings, Sanger sequencing of a large cohort of individuals to directly characterize rs7525979 will be an important confirmation of this initial study. While the *NLRP3* SNP rs7525979 is relatively rare in the population, characterizing its potential role in reducing the risk of PD is important, as this work is among the first steps towards determining whether inflammasome-modulating drugs may be useful for the treatment of PD.

Our finding that *NLRP3* SNP rs7525979 was not associated with SWEDD patients is significant, as it further supports the importance of a specific role for NLRP3 in PD-associated degeneration of DA neurons within the substantia nigra. Patients with SWEDD exhibit symptoms of PD but pathologically do not have evidence of a loss of DA within the brain. In addition to helping to determine the underlying mechanism of DA cell death in classical PD, understanding the function of NLRP3 in DA neurons may help distinguish PD from non-classical parkinsonism. Identifying classical from non-classical PD would greatly improve our ability to stratify patients and thereby potentially improve the sensitivity of our clinical trials with the hope that this would accelerate therapeutic development for PD.

Synonymous SNPs have the capacity to alter the rate of protein translation due to the requirement for a different, and potentially less abundant, tRNA coding for the same amino acid.^53,54^ This has emerged as an important biology, especially in proteinopathies like Huntington’s, AD, and PD,^37,55–57^ because such translational deregulation can underlie protein misfolding that is widely believed to be an important aspect of disease progression. Codon optimality, the translation efficiency of specific codons impacted by relative levels of corresponding tRNAs, thereby creates a layer of regulation over mRNA translation that remains largely unstudied.^53^ Less common codons can disrupt the protein life cycle by impacting the efficiency of translation, which can in turn impact co-translational protein folding,^54,58^ potentially affecting protein function. Increased expression of NLRP3 and molecular chaperone proteins, as observed in our study (Fig. 3), may be the result of altered translation efficiency and disrupted protein folding, which in turn may impact protein function in a variety of ways, including deregulated proteosomal degradation, and/or formation of misfolded protein aggregates.^59^ Our data from the analysis in HEK293 cells enables us to predict that the *NLRP3* SNP rs7525979 reduces the risk of PD by altering the NLRP3 protein life cycle. While these initial mechanistic studies are critical in understanding how novel variants impact protein biology, these results are not sufficient to conclude that the rs7525979 variant will have the same impact on NLRP3 protein in other cell types. For this reason, future studies will focus on the development of genetically engineered neuronal cells and animals designed to rigorously characterize the rs7525979 variant in DA neurons, as well as other cell types of interest, including microglia.^41^

Our combined findings support a model in which the presence of the synonymous variant rs7525979, statistically associated with a reduced risk of PD (Table 1), alters *NLRP3* translation and results in the accumulation of a ubiquitinated, insoluble form of NLRP3, a protein state that is suggestive of protein inactivation.^60^ These findings are consistent with multiple animal models in which loss of *Nlrp3* blocks the development of PD symptomology.^17,18,20^ Our studies provide compelling evidence of the importance of the NLRP3 inflammasome in PD and may provide the basis for novel therapeutic approaches aimed at identifying therapeutic strategies for modulating NLRP3 activity in human neuroinflammatory disorders.^11,61^

## Methods

### Tissue specimens

De-identified postmortem tissues (*n* = 28) were obtained from control donors and patients diagnosed with PD and verified by a board-certified neuropathologist. Tissues were obtained and cataloged by the Department of Pathology at Dartmouth-Hitchcock Medical Center (DHMC, Lebanon, NH) in collaboration with the Wisconsin Parkinson’s Association (WPA, Milwaukee, WI). No genetic analysis was conducted using these tissues. De-identified tissue was deemed “not human subject” as approved by the Institutional Review Board at Dartmouth-Hitchcock Medical Center.

### Immunohistochemistry (IHC)

Immunostains were performed on 5 μM formalin-fixed paraffin-embedded histologic sections using the automated Leica Bond-Max platform. Anti-NALP3/CIAS1 (NLRP3) (Abcam, Cambridge, MA) primary antibodies were detected using a post-primary biotin-free alkaline phosphate polymer and fast red chromogen (Leica Biosystems, Buffalo Grove, IL). Rabbit monoclonal anti-CASP1 antibodies were used for IHC (Cell Signaling Technology, Danvers, MA) and polyclonal rabbit anti-CASP1 anti-sera utilized for SDS-PAGE was generously provided by Dr. Gabriel Nunez (University of Michigan Medical School, Ann Arbor, MI).

### SDS-PAGE and mRNA analysis

SDS-PAGE, immunoblotting, and real-time PCR analyses were performed as previously described.^62,63^ Gel electrophoresis was conducted using the NuPAGE system with 4–12% pre-cast gradient gels and MES/SDS running buffer (Thermo Fisher Scientific, Waltham, MA). For human mesencephalic tissues, primary antibodies for NLRP3 were the same as described above. The following antibodies were used for cell culture lysates and immunoprecipitation assays: anti-NLRP3/NALP3 (Adipogen, San Diego, CA; Cell Signaling Technology, Danvers, MA), anti-c-Myc [9E10] (Abcam, Cambridge, MA), anti-Ubiquitin (Cell Signaling, Danvers, MA), anti-BiP (HSPA5) (Cell Signaling, Danvers, MA), anti-Nurr1 (Santa Cruz Biotechnology, Dallas, TX), anti-tyrosine hydroxylase (Abcam, Cambridge, MA), and anti-actin horseradish peroxidase (HRP)-coupled (Sigma-Aldrich, St. Louis, MO) antibodies. Immunoreactivity was detected using HRP-conjugated anti-rabbit and anti-mouse secondary antibodies (Millipore, Billerica, MA) as appropriate in combination with a chemiluminescent detection method (ECL-Plus, Thermo Fisher Scientific, Waltham, MA). *NLRP3* transcripts were analyzed using real-time PCR with amplification detected using SYBR Green (Bio-Rad Laboratories, Hercules, CA), *NLRP3* forward primer: CACTTCCAGTTTTTGCCGGG, and *NLRP3* reverse primer: GGGAATGGCTGGTGCTCAAT.

### SNP analysis

For data analysis, we used the libraries SeqArray, SeqVarTools,^64^ SNPassoc,^65^ and R version 3.2.2 (R Core Team, 2015). Fisher exact tests indicated that the distribution of genotypes for all four SNPs considered was within Hardy–Weinberg equilibrium for the study population (overall and within each stratum: case, control, and SWEDD). Dominant unconditional logistic regression inheritance models were used to test the risk of PD or SWEDD associated with the SNPs variants adjusted for covariates. A sensitivity analysis additive unconditional logistic regression inheritance model was used to evaluate the chi-squared test for linear trend of the variants.

### Site-directed mutagenesis

DNA corresponding to the human *NLRP3* gene (NM_004895) was purchased from Origene (clone RC220952, Rockville, MD). Site-directed mutagenesis to generate *NLRP3* harboring the rs7525979 variant (*NLRP3*^*979*^) was performed using the Stratagene QuikChange Kit (Agilent Technologies, Santa Clara, CA). Resulting constructs were sequence verified using standard techniques.

### Cell culture

HEK293 cells (ATCC, CRL-1573) were cultured in 10% fetal bovine serum, 1% penicillin/streptomycin in Dulbecco’s modified Eagle’s medium (DMEM) with 4.5 g/L glucose, L-glutamine, and sodium pyruvate (Corning, Corning, NY). Media was replenished every 2–3 days, and all cells were grown in culture incubated at 37 °C supplemented with 5% CO_2_. Cells were transfected with vectors encoding either *NLRP3*^*WT*^ (Origene, Rockville, MD) or *NLRP3*^*979*^ and cultures were independently selected with G418 Sulfate to generate stable, polyclonal cell lines expressing *NLRP3*^*WT*^, *NLRP3*^*979*^, or empty vector control. *NLRP3* constructs were c-terminal epitope tagged with c-Myc and FLAG epitopes. SH-SY5Y cells (ATCC, CRL-2266) were cultured in 10% fetal bovine serum, 1% penicillin/streptomycin in DMEM/Hams F12 50/50 media with L-glutamine (Corning, Corning, NY) at 37 °C supplemented with 5% CO_2_. Cells were differentiated with 10 μM retinoic acid (RA; Sigma-Aldrich, St. Louis, MO) for 7 days, replenishing media with RA every 2 days. LUHMES cells (ATCC, CRL-2927) were cultured according to the supplier’s instructions. Briefly, culture surfaces were pre-coated with 50 μg/mL poly-L-ornithine (Sigma-Aldrich, St. Louis, MO), followed by 1 μg/mL Human Fibronectin (Sigma-Aldrich, St. Louis, MO). Cells were cultured in DMEM/F12 supplemented with 1% N2 (Thermo Fisher Scientific, Waltham, MA), 1% penicillin/streptomycin, and 40 ng/mL basic fibroblast growth factor. LUHMES cells were differentiated onto pre-coated glass coverslips following the published protocols.^66^ To induce differentiation, 24 h following seeding cells were treated with DMEM/F12 media containing 1% N2, 1 μg/mL Doxycycline, 40 ng/mL glial cell-derived neurotrophic factor, 1 mmol/L cAMP (Sigma-Aldrich, St. Louis, MO), and 1% penicillin/streptomycin for 6 days. To induce NLRP3 inflammasome activation, cells were treated with 75 ng/mL LPS for 4 h followed by 10 μM or 20 μM Nigericin (Sigma-Aldrich, St. Louis, MO) treatment for 30 min. Cell lysis buffers include TBS buffer, Triton buffer (150 mM NaCl, 50 mM Tris, 1% Triton), SDS buffer (150 mM NaCl, 50 mM Tris, 1% Triton, 1% SDS), or Urea buffer (8 M Urea, 150 mM NaCl, 50 mM Tris, 1% Triton, 1% SDS) as indicated in the text. All lysis buffers contained protease and phosphatase inhibitors (Sigma-Aldrich, St. Louis, MO). Protein concentrations were determined using a BCA Protein Assay Kit (Thermo Fisher Scientific, Waltham, MA). Immunoprecipitation of NLRP3 was conducted as previously described.^67^

### Immunocytochemistry

Cells were fixed with 10% formalin and permeabilized with 0.1% Triton. Immunostains were conducted with anti-NALP3/CIAS1 (NLRP3) (Abcam, Cambridge, MA), anti-tyrosine hydroxylase (Pel Freez Biologicals, Rogers, AR), anti-β-Tubulin III (Promega, Madison, WI) antibodies and Hoechst stain (Thermo Fisher Scientific, Waltham, MA). Confocal images were obtained and analyzed with the ZEN Software from ZEISS Microscopy.

### Caspase-1 activity assay

Caspase-1 activity was measured using the FAM-FLICA Caspase-1 Assay Kit (ImmunoChemistry Technologies, Bloomington, MN) according to the manufacturer’s instructions.

### Polysome profiling

Translating ribosomes were immobilized with cycloheximide (Sigma-Aldrich, St. Louis, MO) treatment. Transcripts were then separated in a sucrose gradient (10–50%) by ultracentrifugation (30,000 rpm for 4 h). Fractions were isolated with a Gradient Fractionator (BioComp Instruments, Inc., Fredericton, NB Canada) and absorbance of each fraction was measured (Bio-Rad Laboratories, Hercules, CA). mRNA was extracted using affinity columns (Qiagen, Germantown, MD) according to the manufacturer’s instructions, and relative mRNA levels were determined using real-time PCR. Light and heavy polysome fractions were pooled and normalized to total RNA.

### Protein stability and solubility

Cells were treated with cycloheximide (Sigma-Aldrich, St. Louis, MO) for various lengths of time. Lysates were harvested and NLRP3 expression was measured with SDS-PAGE and immunoblotting techniques as described above. To determine protein solubility, serial ultracentrifugation steps were conducted to separate differentially soluble protein fractions. Cells were first collected in TBS buffer, sonicated, and ultracentrifuged at 30,000 × *g* for 30 min at 4 °C. The pellet was then resuspended in Triton Buffer, sonicated, and centrifuged. This process was repeated in increasingly stringent buffers, SDS and Urea respectively, to collect differentially soluble cellular fractions. Following, SDS-PAGE and immunoblotting were conducted to probe for NLRP3 protein.

### Data availability

Data used in the preparation of this article were obtained from the PPMI database (www.ppmi-info.org/data). For up-to-date information on the study, visit www.ppmi-info.org. PPMI—a public–private partnership—is funded by the Michael J. Fox Foundation for Parkinson’s Research and funding partners, including Abbvie, Avid Radiopharmaceuticals, Biogen, Bristol-Myers Squibb, Covance, GE Healthcare, Genentech, GlaxoSmithKline, Lilly, Lundbeck, Merck, Meso Scale Discovery, Pfizer, Piramal, Roche, Servier, and UCB.

## Electronic supplementary material

Supplementary Data

## Electronic supplementary material

**Supplementary information** accompanies the paper on the *npj Parkinson’s Disease* website (10.1038/s41531-018-0061-5).

## References

[1] Pawelec G, Goldeck D, Derhovanessian E (2014). Inflammation, ageing and chronic disease. Curr. Opin. Immunol..

[2] Franceschi C (2000). Inflamm-aging. An evolutionary perspective on immunosenescence. Ann. NY Acad. Sci..

[3] McGeer PL, Itagaki S, Boyes BE, McGeer EG (1988). Reactive microglia are positive for HLA-DR in the substantia nigra of Parkinson’s and Alzheimer’s disease brains. Neurology.

[4] Brochard V (2009). Infiltration of CD4+ lymphocytes into the brain contributes to neurodegeneration in a mouse model of Parkinson disease. J. Clin. Invest..

[5] Mattila KM (2002). Association of an interleukin 1B gene polymorphism (−511) with Parkinson’s disease in Finnish patients. J. Med. Genet..

[6] Gao X, Chen H, Schwarzschild MA, Ascherio A (2011). Use of ibuprofen and risk of Parkinson disease. Neurology.

[7] Nalls MA (2014). Large-scale meta-analysis of genome-wide association data identifies six new risk loci for Parkinson’s disease. Nat. Genet..

[8] Hirsch EC, Hunot S (2009). Neuroinflammation in Parkinson’s disease: a target for neuroprotection?. Lancet Neurol..

[9] Hirsch EC, Vyas S, Hunot S (2012). Neuroinflammation in Parkinson’s disease. Parkinsonism Relat. Disord..

[10] Schroder K, Tschopp J (2010). The inflammasomes. Cell.

[11] Tansey MG, Goldberg MS (2010). Neuroinflammation in Parkinson’s disease: its role in neuronal death and implications for therapeutic intervention. Neurobiol. Dis..

[12] Walsh JG, Muruve DA, Power C (2014). Inflammasomes in the CNS. Nat. Rev. Neurosci..

[13] de Rivero Vaccari JP, Lotocki G, Marcillo AE, Dietrich WD, Keane RW (2008). A molecular platform in neurons regulates inflammation after spinal cord injury. J. Neurosci..

[14] Adamczak SE (2014). Pyroptotic neuronal cell death mediated by the AIM2 inflammasome. J. Cereb. Blood Flow Metab..

[15] Zhang P (2016). Cdk5-dependent activation of neuronal inflammasomes in Parkinson’s disease. Mov. Disord..

[16] Heneka MT (2013). NLRP3 is activated in Alzheimer’s disease and contributes to pathology in APP/PS1 mice. Nature.

[17] Martinez EM (2017). Nlrp3 is required for inflammatory changes and nigral cell loss resulting from chronic intragastric rotenone exposure in mice. Toxicol. Sci..

[18] Yan Y (2015). Dopamine controls systemic inflammation through inhibition of NLRP3 inflammasome. Cell.

[19] Qiao C (2016). Caspase-1 deficiency alleviates dopaminergic neuronal death via inhibiting Caspase-7/AIF pathway in MPTP/p mouse model of Parkinson’s disease. Mol. Neurobiol..

[20] Mao Z (2017). The NLRP3 inflammasome is involved in the pathogenesis of Parkinson’s disease in rats. Neurochem. Res..

[21] Zhou R, Yazdi AS, Menu P, Tschopp J (2011). A role for mitochondria in NLRP3 inflammasome activation. Nature.

[22] Codolo G (2013). Triggering of inflammasome by aggregated alpha-synuclein, an inflammatory response in synucleinopathies. PLoS ONE.

[23] Hauser DN, Hastings TG (2013). Mitochondrial dysfunction and oxidative stress in Parkinson’s disease and monogenic parkinsonism. Neurobiol. Dis..

[24] Bras J, Guerreiro R, Hardy J (2015). SnapShot: genetics of Parkinson’s disease. Cell.

[25] Kumaran R, Cookson MR (2015). Pathways to Parkinsonism Redux: convergent pathobiological mechanisms in genetics of Parkinson’s disease. Human. Mol. Genet..

[26] Lubbe S, Morris HR (2014). Recent advances in Parkinson’s disease genetics. J. Neurol..

[27] Shao QH, Zhang XL, Yang PF, Yuan YH, Chen NH (2017). Amyloidogenic proteins associated with neurodegenerative diseases activate the NLRP3 inflammasome. Int Immunopharmacol..

[28] Meiser J, Weindl D, Hiller K (2013). Complexity of dopamine metabolism. Cell Commun. Signal..

[29] Kalia LV, Lang AE (2015). Parkinson’s disease. Lancet.

[30] Tanner CM (2011). Rotenone, paraquat, and Parkinson’s disease. Environ. Health Perspect..

[31] Braak H, Sandmann-Keil D, Gai W, Braak E (1999). Extensive axonal Lewy neurites in Parkinson’s disease: a novel pathological feature revealed by alpha-synuclein immunocytochemistry. Neurosci. Lett..

[32] Lotharius J (2005). Progressive degeneration of human mesencephalic neuron-derived cells triggered by dopamine-dependent oxidative stress is dependent on the mixed-lineage kinase pathway. J. Neurosci..

[33] Parkinson Progression Marker Initiative The Parkinson Progression Marker Initiative (PPMI). Prog. Neurobiol. 95, 629–635 (2011). 10.1016/j.pneurobio.2011.09.005 PMC9014725 21930184

[34] Komar, A. A. (ed) *Single Nucleotide Polymorphisms: Methods and Protocols* (Humana Press, New York, NY, 2009).

[35] Chasse H, Boulben S, Costache V, Cormier P, Morales J (2017). Analysis of translation using polysome profiling. Nucleic Acids Res.

[36] Casas C (2017). GRP78 at the centre of the stage in cancer and neuroprotection. Front Neurosci..

[37] Maheshwari M (2014). Deficiency of Ube3a in Huntington's disease mice brain increases aggregate load and accelerates disease pathology. Hum. Mol. Genet..

[38] de Lau LM, Breteler MM (2006). Epidemiology of Parkinson’s disease. Lancet Neurol..

[39] Rocha NP, de Miranda AS, Teixeira AL (2015). Insights into neuroinflammation in Parkinson’s disease: from biomarkers to anti-inflammatory based therapies. Biomed. Res. Int..

[40] Zhou K, Shi L, Wang Y, Chen S, Zhang J (2016). Recent advances of the NLRP3 inflammasome in central nervous system disorders. J. Immunol. Res..

[41] Sarkar S (2017). Mitochondrial impairment in microglia amplifies NLRP3 inflammasome proinflammatory signaling in cell culture and animal models of Parkinson’s disease. NPJ Parkinsons Dis..

[42] Banjara M, Ghosh C (2017). Sterile neuroinflammation and strategies for therapeutic intervention. Int J. Inflam..

[43] Davis BK, Wen H, Ting JP (2011). The inflammasome NLRs in immunity, inflammation, and associated diseases. Annu. Rev. Immunol..

[44] Opipari A, Franchi L (2015). Role of inflammasomes in intestinal inflammation and Crohn’s disease. Inflamm. Bowel Dis..

[45] Meng DM (2013). Polymorphisms in the NLRP3 gene and risk of primary gouty arthritis. Mol. Med. Rep..

[46] Day TG (2008). Autoinflammatory genes and susceptibility to psoriatic juvenile idiopathic arthritis. Arthritis Rheum..

[47] Tan MS (2013). NLRP3 polymorphisms are associated with late-onset Alzheimer’s disease in Han Chinese. J. Neuroimmunol..

[48] Lin MK, Farrer MJ (2014). Genetics and genomics of Parkinson’s disease. Genome Med..

[49] van der Brug MP, Singleton A, Gasser T, Lewis PA (2015). Parkinson’s disease: from human genetics to clinical trials. Sci. Transl. Med..

[50] Koonin EV, Aravind L (2000). The NACHT family - a new group of predicted NTPases implicated in apoptosis and MHC transcription activation. Trends Biochem. Sci..

[51] Farasat S, Aksentijevich I, Toro JR (2008). Autoinflammatory diseases: clinical and genetic advances. Arch. Dermatol..

[52] Johnston HR, Hu Y, Cutler DJ (2015). Population genetics identifies challenges in analyzing rare variants. Genet. Epidemiol..

[53] Chen CA, Shyu AB (2017). Emerging themes in regulation of global mRNA turnover in cis. Trends Biochem. Sci..

[54] Chaney JL, Clark PL (2015). Roles for synonymous codon usage in protein biogenesis. Annu Rev. Biophys..

[55] Sweeney P (2017). Protein misfolding in neurodegenerative diseases: implications and strategies. Transl. Neurodegener..

[56] Montgomery SL (2013). Chronic neuron- and age-selective down-regulation of TNF receptor expression in triple-transgenic Alzheimer disease mice leads to significant modulation of amyloid- and Tau-related pathologies. Am. J. Pathol..

[57] Burre J, Sharma M, Sudhof TC (2015). Definition of a molecular pathway mediating alpha-synuclein neurotoxicity. J. Neurosci..

[58] O’Brien EP, Ciryam P, Vendruscolo M, Dobson CM (2014). Understanding the influence of codon translation rates on cotranslational protein folding. Acc. Chem. Res.

[59] Bali V, Bebok Z (2015). Decoding mechanisms by which silent codon changes influence protein biogenesis and function. Int J. Biochem Cell Biol..

[60] Barrett PJ, Greenamyre JT (2015). Post-translational modification of α-synuclein in Parkinson's disease. Brain Res..

[61] Coll RC (2015). A small-molecule inhibitor of the NLRP3 inflammasome for the treatment of inflammatory diseases. Nat. Med..

[62] Havrda MC (2008). Id2 is required for specification of dopaminergic neurons during adult olfactory neurogenesis. J. Neurosci..

[63] Havrda MC (2014). Id2 mediates oligodendrocyte precursor cell maturation arrest and is tumorigenic in a PDGF-rich microenvironment. Cancer Res..

[64] Zheng X (2012). A high-performance computing toolset for relatedness and principal component analysis of SNP data. Bioinformatics.

[65] Gonzalez JR (2007). SNPassoc: an R package to perform whole genome association studies. Bioinformatics.

[66] Zhang XM, Yin M, Zhang MH (2014). Cell-based assays for Parkinson’s disease using differentiated human LUHMES cells. Acta Pharmacol. Sin..

[67] Sullivan JM (2016). Phosphorylation regulates Id2 degradation and mediates the proliferation of neural precursor cells. Stem Cells.

