# Supplementary material for: NLRP3 expression in mesencephalic neurons and characterization of a rare NLRP3 polymorphism associated with decreased risk of Parkinson’s disease
Source: NPJ Parkinsons Dis. 2018 Aug 15;4:24. doi: 10.1038/s41531-018-0061-5 (PMC6093937; doi:10.1038/s41531-018-0061-5)
Supplement: Supplementary file 1 — Supplementary Data [file 41531_2018_61_MOESM1_ESM.pdf]

Supplementary Figures

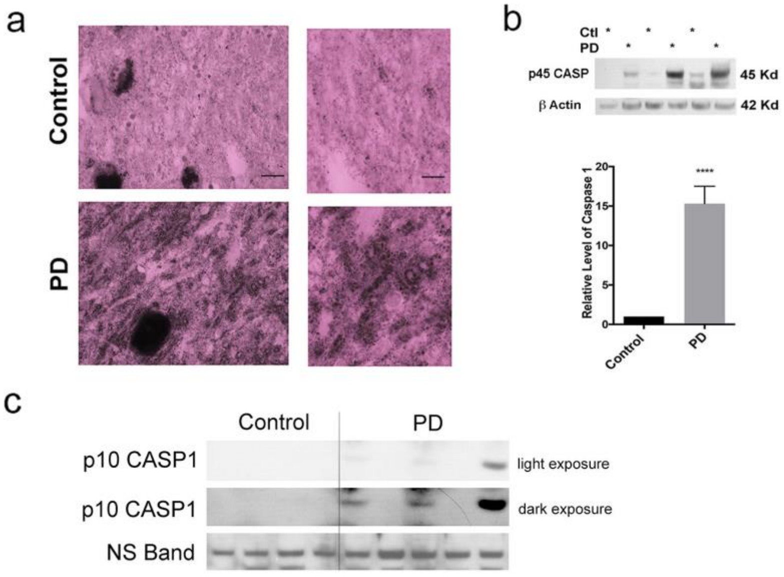

Supplementary Figure 1: Caspase 1 expression and activation in the mesencephalon. **A.** Immunohistologic sections obtained from the human mesencephalon of controls and PD patients were stained with anti-caspase-1 antibodies. **B.** Cleared brain homogenates were prepared from cryopreserved tissues obtained from representative control and PD patients, size fractionated and immunoblotted for caspase-1. Immunoblotting revealed an increase in the 45 kDa caspase-1 protein in PD patients as compared with controls (B, lower panel, n = 3 per group, P < 0.001). **C.** In a subset of extracts from PD patients, a 10 kDa caspase-1 immunoreactive band was observed indicative of caspase-1 cleavage and activation. β-actin immunoblotting was conducted to ensure accuracy of protein loading.

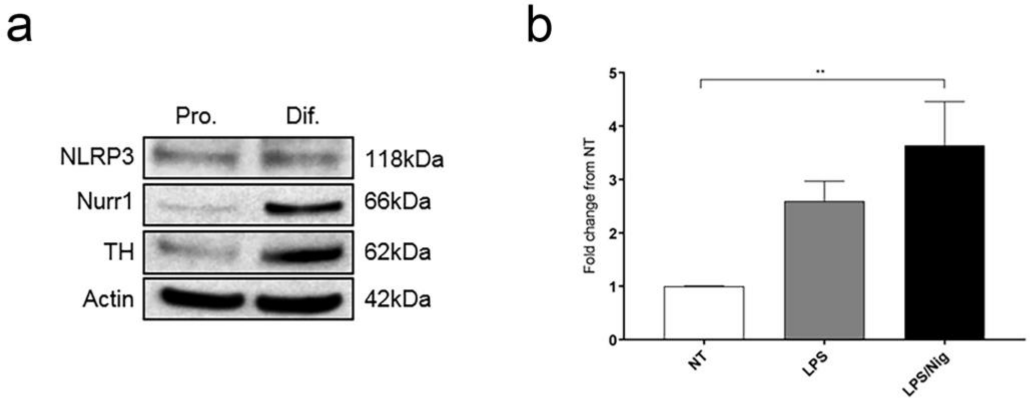

Supplementary Figure 2: NLRP3 inflammasome expression and activity in SH-SY5Y cells.

A. Undifferentiated (Pro.) and differentiated (Dif.) SH-SY5Y cells were harvested and interrogated with SDS-PAGE and immunoblotting. Membranes were probed with anti-NLRP3, anti-Nurr1, anti-TH and anti-actin antibodies. **B.** Differentiated SH-SY5Y cells were treated with LPS or LPS+Nigericin and subjected to a FAM-FLICA caspase-1 assay. Caspase-1 activity was statistically elevated upon treatment (** $P < 0.01$, one-way ANOVA, $n = 3$ biological replicates).

Supplementary Table

Supplementary Table 1. Parkinson disease risk in the Parkinson's Progression Markers Initiative (PPMI) study associated with the *NLRP3* SNP rs7525979 (additive inheritance model)

	**Cases n=402, n (%)**	**Controls n=182, n (%)**	**OR (95% CI)**	***P*-value**
Age, mean (sd)	62.2 (9.7)	61.6 (11.0)	1.005 (0.99 - 1.02)	0.55
Sex				
Male	260 (64.7)	116 (63.7)	1.0 (Reference)	
Female	142 (35.3)	66 (36.3)	0.94 (0.65 - 1.37)	0.76
rs7525979 (C/C)				
C/C (0)	357 (88.8)	148 (81.3)	1.0 (Reference)	
C/T (1) + T/T (2)	45 (11.2)	34 (18.7)	0.59 (0.38 - 0.93)	0.02

Model adjusted for all variables in the table
